# Barriers and enablers to physical activity behaviour in older adults during hospital stay: a qualitative study guided by the theoretical domains framework

**DOI:** 10.1186/s12877-022-02887-x

**Published:** 2022-04-10

**Authors:** Hanneke C. van Dijk - Huisman, Petra H. Raeven-Eijkenboom, Fabienne J. H. Magdelijns, Judith M. Sieben, Robert A. de Bie, Antoine F. Lenssen

**Affiliations:** 1 Department of Physiotherapy, Maastricht University Medical Centre, P.O. Box 5800, 6202 AZ Maastricht, The Netherlands; 2 CAPHRI School for Public Health and Primary Care, Maastricht University, P.O. Box 616, 6200 MD Maastricht, The Netherlands; 3 Avans University of Applied Sciences, P.O. Box 90.116, 4800 RA Breda, The Netherlands; 4 Department of Internal Medicine, division of General Medicine and Clinical Geriatric Medicine, Maastricht University Medical Centre, P.O. Box 5800, 6202 AZ Maastricht, The Netherlands; 5 Department of Anatomy & Embryology, Maastricht University, P.O. Box 616, 6200 MD Maastricht, The Netherlands; 6 Department of Epidemiology, Maastricht University, P.O. Box 616, 6200 MD Maastricht, The Netherlands

**Keywords:** Physical activity behaviour, Hospital, Older adults, Barrier, Enabler, Theoretical domains framework

## Abstract

**Background:**

Older adults admitted with an acute medical illness spent little time active during hospitalisation and this has been associated with negative health outcomes. Understanding which barriers and enablers influence the physical activity behaviour of hospitalised older adults is a first step towards identifying potentially modifiable factors and developing, evaluating and implementing targeted interventions aimed at increasing their physical activity behaviour. Using a theoretical framework has been found to be more successful in changing behaviour than using a non-theory driven approach. This study aimed to explore barriers and enablers to physical activity behaviour in older adults admitted to a hospital with an acute medical illness, as perceived by patients and healthcare professionals, and to categorise them using the Theoretical Domains Framework (TDF).

**Methods:**

A qualitative study was conducted at a combined university and regional hospital in the Netherlands between January 2019 and February 2020. Older adults (≥70 years) admitted with an acute medical illness, and healthcare professionals (nurses, physicians, physiotherapists) were recruited using purposive sampling. Semi-structured interviews were audiotaped, transcribed and analysed using directed qualitative content analysis. Barriers and enablers to physical activity behaviour during hospitalisation were identified and coded using the TDF.

**Results:**

Meaning saturation was determined after interviews with 12 patients and 16 healthcare professionals. A large number of barriers and enablers were identified and each categorised to 11 of the 14 domains of the TDF. The ‘*Environmental Context and Resources*’ domain in particular yielded many examples, and revealed that the hospital environment exerts an inactivating influence on patients.

**Conclusions:**

The large number of identified barriers and enablers highlights the complexity of influencing older adults’ physical activity behaviour during hospitalisation. This overview of barriers and enablers to physical activity behaviour in older adults admitted to a hospital with an acute medical illness represents an initial step towards developing, evaluating and implementing theory-informed behaviour change interventions to improve hospitalised older adults’ physical activity levels. It can assist clinicians and researchers in selecting modifiable factors that can be targeted in future interventions.

**Supplementary Information:**

The online version contains supplementary material available at 10.1186/s12877-022-02887-x.

## Introduction

Older adults admitted to hospital with an acute medical illness spend little time active. They spend an average of 30 to 117 min a day standing or walking; the remainder of the day is spent lying in bed or sitting in a chair [1–8]. Regardless of illness severity or comorbidities, this inactive behaviour is strongly associated with functional decline, increased length of hospital stay, increased risk of institutionalisation, and mortality [1, 2, 4, 5, 8–15]. These older adults are already at high risk of negative outcomes due to a high prevalence of multi-morbidity and age-related impairments, such as a decreased physiological reserve capacity, malnutrition, cognitive impairment, incontinence, and sensory impairment [16]. Therefore, interventions aimed at improving the physical activity (PA) behaviour of older adults during hospitalisation are needed to improve patient outcomes [17].

Such interventions are more likely to be effective if they are designed to target underlying factors that influence behaviour [18]. Understanding which barriers and enablers influence the PA behaviour of hospitalised older adults is a first step towards identifying potentially modifiable factors and developing, evaluating and implementing targeted interventions [18–20].

Several studies have investigated barriers and enablers to PA behaviour during hospital stay for acute care in older adults, from the perspectives of patients [17, 21], healthcare professionals (HCPs) [22–24] or both [25–27]. Brown et al. were the first to explore barriers to PA from the perspectives of patients and HCPs. They identified patient-related barriers (e.g., symptoms), treatment-related barriers (e.g., bed rest orders), institutional barriers (e.g., availability of assistance) and attitudinal barriers (e.g., concerns about falling) [27]. Following studies identified additional barriers and enablers, such as HCPs prioritising other care processes over promoting PA [22, 24, 25], patients being attached to IV-poles or other medical devices [22, 25], HCPs’ lack of training on how to safely mobilise hospitalised patients [23], patients receiving encouragement from HCPs [17], and patients wanting to prevent the negative effects associated with bed-rest [17].

The use of theories of behaviour or behaviour change has been found to be more successful in changing behaviour than using a non-theory driven approach [28, 29]. As the Medical Research Council guidance on the development of complex interventions advocates the use of theory to identify barriers and enablers to behaviour change [30], our study expanded on previous studies by adopting a theoretical framework to categorise barriers and enablers. As a large number of theories of behaviour and behaviour change have been developed, selecting an appropriate theory can be challenging. The Theoretical Domains Framework (TDF) is an overarching theoretical framework in which constructs of 33 theories of behaviour and behaviour change are integrated and simplified into 14 domains [31]. It is developed to make theories more accessible and was chosen for this study due to its inclusive nature, incorporating cognitive, affective, social and environmental influences on behaviour [29]. It enables a systematic and theory driven approach to categorise barriers and enablers into the fourteen domains. Moreover, it can guide the development of interventions by linking potentially modifiable factors to intervention functions and behaviour change techniques (BCTs) [29, 31–34]. The objective of our study was to explore and categorise patient- and HCP-perceived barriers and enablers to PA behaviour in older adults admitted to a hospital with an acute medical illness, using the TDF.

## Methods

### Study design

This qualitative study used semi-structured interviews to explore barriers and enablers to PA behaviour in older adults admitted to hospital with an acute medical illness. Directed qualitative content analysis was performed using the TDF, and overarching themes were identified within each domain. This study was conducted at the Department of Internal Medicine of Maastricht University Medical Centre (MUMC+) in Maastricht, the Netherlands, between January 2019 and February 2020. The consolidated criteria for reporting qualitative research (COREQ) were applied to report this study (Additional file 1) [35].

### Setting and research group

The MUMC+ is a 715-bed, combined university and regional hospital. The Department of Internal Medicine can accommodate 33 patients with a nurse to patient ratio of 1:6. Patients are admitted to single, double, or four-bed rooms. The ward has a communal ‘living room’ and an exercise room. Nursing assistants, nutritionists and physiotherapists help mobilise, feed, and monitor patients. On weekdays, the physiotherapist is present at the ward for 2.5 h.

The research group consisted of one health scientist (JMS), four health scientists with a background as physiotherapists (HCvDH, PHRE, AFL, RAdB), and one geriatrician (FJHM). HCvDH and AFL were involved in a care programme to improve the PA behaviour of patients during hospital stay. HCvDH and FJHM worked at the ward and had been treating some of the patients.

### Participant selection and recruitment

Patients were sampled purposively by consulting HCPs, in order to include a variety of medical conditions, sex and age. Older adults admitted to the Department of Internal Medicine at MUMC+ were recruited by their attending physician and were asked to consent to being contacted by the researcher (HCvDH). Patients received verbal and written information about the study from the researcher within 48 h after admittance. Patients were contacted again 3 days later, and written informed consent was obtained before study initiation.

Eligible patients were included if they were 70 years or older, had been admitted with an acute medical illness, were able to verbally communicate in Dutch (i.e., no language barrier or aphasia), had been living at home before hospitalisation, and had been able to walk independently on level surface with or without a walking aid 2 weeks before admission, as reported using the Functional Ambulation Categories (FAC > 3) [36, 37]. Patients were excluded if the attending physician had reported in the electronic medical record that patients had contraindications to walking, were mentally incapacitated, were unable to be interviewed due to cognitive problems or severe agitation, or had a life expectancy of less than 3 months. In case of any doubt, patients were not considered eligible. Patients who had previously participated in this study were excluded as well.

For every included patient, an HCP involved with this patient was also recruited. Nurses, physiotherapists and physicians (including resident physicians) who had been working at the hospital for more than 1 month were eligible. HCPs were sampled purposively by the researcher to include a variety of professions, work experience, sex and age. HCPs received verbal and written information about the study from the researcher. They were contacted again 3 days later and written informed consent was obtained before study initiation. Confidentiality of data processing and pseudonymisation of participants were guaranteed.

### Data collection

Semi-structured, face-to-face individual interviews were conducted by two researchers (HCvDH, PHRE) experienced in qualitative research. Patients were interviewed on day 4–6 after admission, thus ensuring they had had sufficient time to experience barriers and enablers to PA behaviour during their hospitalisation. HCPs were interviewed within 7 days of the patient interview. A semi-structured interview approach was used with open questions. Separate interview guides were created for patients (Additional file 2) and HCPs (Additional file 3) based on existing literature on barriers and enablers to PA behaviour [17, 27, 38, 39]. To provide maximum opportunity for the participants to answer in their own words and direction, the interview guides were not based on the domains of the TDF. The interview guides were piloted by interviewing one patient and three HCPs. Pilot interviews were not included in the final sample. Interviews were conducted by two different interviewers to avoid bias by a single interviewer’s style and to enhance confirmability. Before the first interviews started, the interviewers discussed their own views on barriers and enablers to PA behaviour during hospitalisation, with the aim of increasing awareness of feelings and opinions. Interviews were conducted in a separate room at the ward to guarantee privacy. They were expected to last approximately 30 min and were audiotaped. When starting the interview, the interviewers introduced themselves and briefly explained the objectives of the interview. Notes were taken during the interview to capture observations and initial interpretations. Member checking was performed by providing a verbal summary of the interview to the participant. Interviews were transcribed full verbatim and reviewed by two researchers (HCvDH, PHRE) to verify content. Participants were offered the opportunity to read the transcript, to offer comments or additional thoughts.

Demographic data was collected at the start of the interview. Patients were asked for their age, sex, ability to walk independently on the day of the interview (yes/no), type of walking aid used on the day of the interview, whether they had received one or more physiotherapy sessions during hospitalisation (yes/no) and the amount and type of PA they had performed in the 2 weeks prior to hospitalisation. HCPs were asked for their age, sex, profession, and how long they had been working at the MUMC+ Department of Internal Medicine.

### Theoretical domains framework

The validated TDF comprises 84 theoretical constructs from 33 theories of behaviour and behaviour change, embedded in 14 domains: *Knowledge*; *Skills*; *Social/Professional Role and Identity*; *Beliefs About Capabilities*; *Optimism*; *Beliefs about Consequences*; *Reinforcement*; *Intentions*; *Goals*; *Memory, Attention and Decision Processes*; *Environmental Context and Resources*; *Social Influences*; *Emotion*; *and Behavioural Regulation* [29, 40]. The TDF has been applied across a wide range of healthcare settings and clinical behaviours [29, 31]. It has been extensively used as a guide to identify and categorize barriers and enablers to PA in other populations and settings such as at school [41], at work [42], among older adults living with HIV [43], in asylum seekers [44], and in stroke survivors [45].

### Data analysis

Directed qualitative content analysis as described by Hsieh and Shannon was used as an approach to organise and analyse the data [46]. An a-priori coding template was developed based on the 14 domains of the TDF. Analysis started after two interviews had been completed. Two researchers (HCvDH and PHRE) independently read and re-read each transcript and coded barriers and enablers using the TDF domains. The theoretical definitions and component constructs of the domains, as presented in Additional file 4, were used to guide the coding process. Barriers and enablers were coded separately, and were assigned to more than one domain if the content suited multiple domains. The researchers discussed coding discrepancies and iteratively modified the coding template after every two interviews. Any discrepancies resulted in reviewing the data and codes. If necessary, a senior researcher (AFL) was consulted to discuss and resolve disputes. An audit trail was created and new or changed codes were documented during every iteration, including a definition of each code and a description of the issues it captured. Emerging themes were explored in subsequent interviews and this process was repeated until a final coding template had been developed and meaning saturation was observed by the researchers. Meaning saturation was defined as the point where a full understanding was created of the issues, and when no further dimensions, nuances, or insights of issues were found [47]. This was assessed separately for patients and HCPs. Two additional interviews were conducted per group to ensure that no new information was discovered and saturation was achieved, after which inclusion of patients or HCPs ended. Confirmability of the outcomes was enhanced by creating an audit trail. NVivo (QSR International Pty Ltd. [2018] NVivo [Version 12]) software was used to facilitate data analysis.

Finally, a descriptive summary of the reported barriers and enablers was composed for each TDF domain. Quotes from participants’ transcripts were included as illustrations. Dutch quotes and code labels were translated into English and checked by a professional translator/editor for correct conveyance of their meaning [48].

## Results

### Participants

Thirty potential participants were approached. Twenty-eight of them (12 patients, 6 nurses, 5 physiotherapists, and 5 physicians) were included in the study. Two patients declined participation. Meaning saturation occurred after 10 patient and 14 HCP interviews. Subsequent interviews did not provide additional information regarding the understanding of issues, resulting in a total of 12 patient and 16 HCP interviews. Characteristics of patients and HCPs are summarised in Tables 1 and 2, respectively. The median (interquartile range [IQR]) age of the patients was 83 (75–85) years and 7 patients (58.3%) were male. The duration of the patient interviews ranged from 27 to 57 min. The median (IQR) age of the HCPs was 27 (24-33) years and 3 HCPs (18.8%) were male. The duration of the HCP interviews ranged from 33 to 61 min.

**Table 1:** Patient characteristics

Patient code	Sex	Age (years)	Ability to walk independently on the day of the interview	Walking aid	Received PT during hospital stay	PA performed 2 weeks prior to admission
P0001	Male	84	Yes	Walker	Yes	-Walking in and around the house (50–60 m.)
P0002	Female	71	Yes	Walker	Yes	-Fitness (1 h per week) -Walking (< 1 km) -Cycling to grocery store -Volunteer work at elderly home
P0003	Male	83	Yes	Walker	Yes	-Walking inside the house
P0004	Male	82	Yes	Walker	No	-Walking inside the elderly home -Dancing (behind the walker) -Performing activities of daily living
P0005	Male	85	Yes	None	No	-Nordic walking (5 km) -Volunteer work (walking, repairing bicycles) -Doing groceries
P0006	Female	82	Yes	Walker	Yes	-Walking (30 min per day) -Performing household activities
P0007	Male	91	Yes	Cane	No	-Walking (once per week, 1 km) -Doing groceries -Performing household activities
P0008	Female	75	Yes	Walker	No	-No physical activity performed, only lying in bed
P0009	Female	75	Yes	None	Yes	-Performing household activities
P0010	Male	87	Yes	None	Yes	-Walking (200-300 m. per day) -Performing household activities
P0011	Male	83	Yes	None	Yes	-Walking to grocery store (350 m.) -Performing household activities
P0012	Female	73	No	Walker	Yes	-Swimming (once per week) -Walking to grocery store (twice per week, < 1 km.) -Following an exercise program on the television

*PT* Physiotherapy, *PA* Physical activity

**Table 2:** Healthcare professional characteristics

Healthcare professional code	Sex	Age (years)	Profession	Work experience (months / years)
H3001	Female	28	Physiotherapist	6 months
H2002	Male	27	Physician	1 year
H1003	Female	25	Nurse	8 years
H2004	Female	24	Physician	4 months
H2005	Female	24	Physician	3 years
H1006	Female	25	Nurse	4 years
H1007	Female	27	Nurse	3 years and 6 months
H2008	Female	23	Physician	4 months
H3009	Female	27	Physiotherapist	3 years and 11 months
H2010	Female	33	Physician	8 years and 7 months
H3011	Female	32	Physiotherapist	10 years
H3012	Female	25	Physiotherapist	2 years and 6 months
H1013	Male	48	Nurse	13 years and 11 months
H1014	Female	54	Nurse	36 years
H3015	Female	35	Physiotherapist	12 years and 6 months
H1016	Male	24	Nurse	4 years

### Theoretical domains framework

**Fig. 1:**
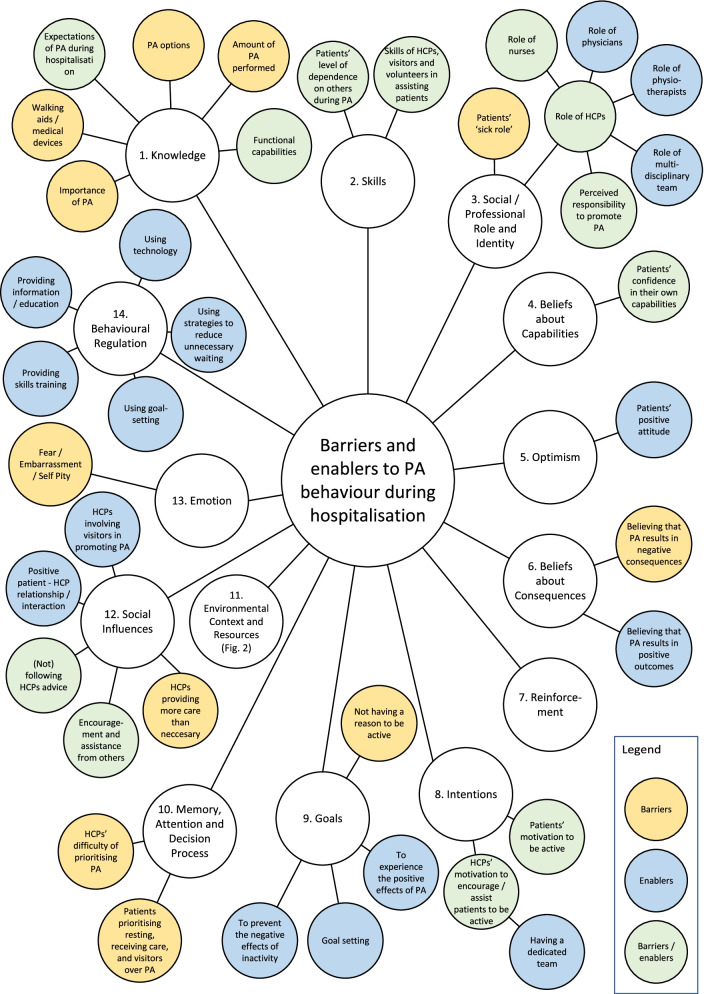
Visualisation of the TDF coding of barriers and enablers to PA behaviour in hospitalised older adults. TDF = Theoretical Domains Framework, PA = physical activity, HCP = healthcare professional

A complete overview and a visualisation of the TDF coding of all barriers and enablers are provided in Additional file 5 and Fig. 1, respectively. No barriers were assigned to the domains of *Optimism, Reinforcement,* and *Behavioural Regulation*. No enablers were assigned to the domains of *Reinforcement, Memory, Attention and Decision Process,* and *Emotion.*

#### 1. Knowledge

Patients and HCPs described that patients, visitors, and HCPs are not always aware that it is important for patients to stay active during hospitalisation, or what patients could do to stay active. HCPs suggested explaining expectations regarding PA to patients and visitors prior to admission. Patients and HCPs also perceived a lack of knowledge of patients’ functional capabilities as a barrier, while having this knowledge was perceived as an enabler.

H1006: ‘Often you don’t know to what extent patients are able to stand. And that’s the reason why we didn’t *get* patients like this one out of bed.’

Other barriers described were a lack of knowledge about walking aids and opportunities to be active despite being attached to medical devices (e.g., an IV-pole).

#### 2. Skills

Being dependent on HCPs or visitors during PA was reported as a barrier by patients and HCPs, while being independent was reported as an enabler. Main reasons for being dependent were reduced physical capabilities, perceived risk of falls, and cognitive impairments.

*H2004: ‘He wasn’t even able to get out of bed and stand-up without assistance. And so I think feeling insecure about walking is holding him back, the fact that his fall risk would increase if he did it alone, so he’s actually dependent on someone else to become active.’*

HCPs identified a lack of skills to assist patients during PA as a barrier, while having these skills was identified as an enabler. Nurses questioned whether all volunteers and visitors have these skills.

#### 3. Social/professional role and identity

HCPs described that older adults may adopt a ‘sick role’ during hospitalisation and that these patients feel that they do not need to be active. They believe that they should remain in bed and often adopt a more dependent attitude than necessary.

*H1014: ‘In general, I think older people always assume they have to stay in bed because they are sick.’*

Patients and HCPs also identified different professional roles within a multidisciplinary team as enablers (Table 3). The status of a physician was mentioned by many HCPs as an important influence on patients’ decision to become active.

**Table 3:** Social/Professional roles of different professions identified as enablers

Profession	Role	Reported by			
		P	N	PH	PT
Physician	Status of a physician		x	x	x
	Providing patients with information on the importance of PA during hospitalisation		x	x	
	Referring patients to therapy services		x	x	x
Nurse	Providing patients with information on the importance of PA during hospitalisation		x	x	
	Assessing functional capabilities		x	x	x
	Providing encouragement and assistance during PA		x		x
	Providing walking aids		x	x	
	Training other nurses in safe patient handling and mobility		x		
Physiotherapist	Providing patients with information on the importance of PA during hospitalisation	x	x	x	x
	Assessing functional capabilities and risk of inactive behaviour		x	x	x
	Assessing fall risk and fear of falling			x	x
	Providing encouragement and assistance during PA		x		x
	Providing walking aids			x	x
	Instructing group exercise classes		x		x
	Providing supervision in exercise rooms	x			x
	Stimulating prevention				x
	Coaching patients, visitors and other HCPs	x	x	x	x
	Educating other HCPs on the importance of PA during hospitalisation				x
	Training other HCPs in safe patient handling and mobility		x		x

*P* Patient, *N* Nurse, *PH* Physician, *PT* Physiotherapist, *PA* Physical activity, *HCP* Healthcare professional

*H1006:* ‘*When they hear it from their physician they jump out of bed, and when they hear it from us [nurses] it’s: “oh girl, why don’t you just go to the next patient”.’*

Moreover, having a dedicated multidisciplinary team in which everyone perceives a responsibility to promote PA behaviour was seen as important. However, nurses, physicians and physiotherapists also described that not all HCPs perceive a responsibility to promote PA behaviour. They explained that this responsibility is often attributed to other disciplines, mostly physiotherapists.

#### 4. Beliefs about capabilities

Patients and HCPs described that patients who lack confidence in their own functional capabilities are less active than those who are confident about them.

*P0011: ‘Feeling insecure about falling... not being stable enough... That’s what I fear most... and then I think “I hope I don’t fall, I hope I don’t get too dizzy...” These thoughts shouldn’t come into my mind, but they do.’*

#### 5. Optimism

No barriers emerged within this domain. Both patients and HCPs described that patients who adopt a positive attitude are more likely to be active.

*P0002: ‘I walk my usual round every day because it’s in my nature. But then I think, people who don’t do that ... get up, just walk one or two rounds. A little further each day. Yes, also to ensure that you get out of that vicious circle of “I’m not able to do it”. And don’t think about it too long, you must make sure that you get better, don’t dwell on it. But again, I’m a very positive person, fortunately.’*

#### 6. Beliefs about consequences

Many patients described that they were active because they believed that PA stimulated their recovery and physical fitness.

*P0005: ‘I love being active. I just wanted to be fit. And you can’t do that if you’re lying down.’*

Two nurses described that they preferred patients to remain at the ward because otherwise they would not be able to reach them.

#### 7. Reinforcement

No barriers or enablers emerged within this domain.

#### 8. Intention

Nearly all participants discussed patients’ motivation. Many patients felt motivated to engage in PA to prevent the negative effects of inactivity or to experience the positive effects of PA. However, feeling sick, being delirious, not perceiving the need to be active, not wanting to leave the ward, and a lack encouragement from others were reported to decrease patients’ motivation. HCPs also discussed their own motivation to encourage and assist patients. Having a dedicated team, motivated to encourage and assist patients during PA was perceived as an enabler.

*H1007: ‘We really are a department where patients are mobilised in a chair after the morning care round. In the afternoon they are allowed to lie in bed for an hour, but after that they have to get out of bed again. We really are a department that gets everyone out of bed or motivates them to walk to the living room, instead of using a wheelchair.’*

However, they also explained that their motivation was closely associated with their workload.

*H1014: ‘When we have little time it’s just faster to take someone to the living room in a wheelchair. Usually this is caused by lack of time. However, many people would actually be able to walk there.’*

#### 9. Goals

Patients and HCPs explained that patients need a reason to get out of bed, walk around or exercise. However, purposeful, meaningful activities are often lacking during a hospital stay. To prevent sedentary behaviour, they suggested using goalsetting and providing patients with targets to accomplish.

*P0005: ‘You are active because you have a goal, right. At home you go to the shop or do some cleaning or whatever. You don’t have that here of course; you don’t have a specific goal here.*

Wanting to prevent the negative effects associated with inactivity (e.g., functional decline, becoming dependent on others, pulmonary complications, pressure ulcers, pain and stiffness, deep vein thrombosis, and mortality) was described by patients and HCPs as a reason to either become active or to promote PA behaviour. Patients additionally described wanting to experience the positive effects of PA (e.g., recovery, regaining physical fitness, regaining self-confidence, going home), as well as preventing boredom, seeking distraction, wanting to smoke, and wanting privacy.

#### 10. Memory, attention and decision process

Although no enablers emerged within this domain, the difficulty of prioritising PA during hospitalisation was often discussed as a barrier. Patients and HCPs reported that patients tend to prioritise care rounds, examinations, visitors and resting over PA. HCPs also explained that lack of time, insufficient staffing, or having high acuity patients at the ward often led to other tasks getting priority over promoting patients’ PA behaviour, even though they were aware of the importance of it.

*H1006: ‘I was the only [registered] nurse for these patients and I had an intern with me. Because I was busy with so many things, I didn’t get a chance to stimulate other patients [to be active]. Yes, and then you have to set priorities. What’s more important? Someone with a life-threatening situation like today...’*

#### 11. Environmental context & resources

**Fig. 2:**
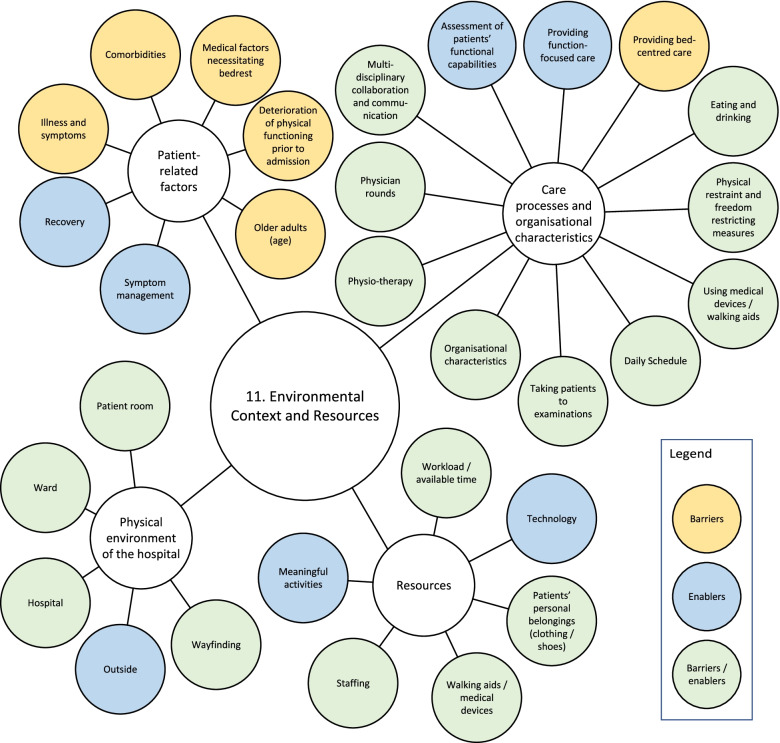
Visualisation of the TDF coding of barriers and enablers to the domain of *Environmental Context and Resources*. TDF = Theoretical Domains Framework

A visualisation of the TDF coding of all barriers and enablers into the domain of *Environmental Context and Resources* is provided in Fig. 2.

##### Care processes and organisational characteristics

HCPs described that many different care processes are concentrated around the hospital bed, such as physician rounds or medication rounds. This ‘bed-centred care’ is thought to have an inactivating effect on patients, while care processes aimed at maintaining or improving independence in activities of daily living (i.e., providing ‘function-focused care’) were described as activating.

*P0011: ‘They shouldn’t bring meals to the bed. Yes, of course it’s easier if I get it in bed, but eating should be done at a table. You can eat at a table, but it’s not required. They should say: “Your dinner is ready at the table or in the hallway, you should go and get it”.’*

HCPs described that many patients are not aware of their daily schedule and stay in their room because they are afraid to miss HCPs. Providing a structured daily schedule with care processes scheduled at predefined times was suggested to reduce unnecessary waiting and create more time for PA. Other barriers reported by patients and HCPs are using physical restraint and freedom-restricting measures, as well as being attached to IV-poles, drains, or urinary catheters. As a solution, HCPs proposed minimising their use, or facilitating PA despite these restrictions. Furthermore, care processes related to communication and collaboration between nurses, physiotherapists, and physicians were described by HCPs as either a barrier or an enabler.

##### Patient-related factors

Medical factors and ageing were identified as barriers by many patients and HCPs. Illness and accompanying symptoms (i.e., fatigue, pain, dizziness, weakness, reduced exercise capacity, dyspnoea, poor balance, hypotension, nausea, oedema, diarrhoea) were reported many times, but deterioration of physical functioning prior to hospitalisation, comorbidities (e.g., cognitive problems, obesity, visual and hearing impairments) and medical conditions requiring bedrest were also mentioned.

*H1016: ‘Being ill is just the biggest barrier. Not feeling well and being in pain. I think pain is a very important factor. If the pain is bearable then patients are more likely to get out of bed.’*

By contrast, recovery and managing symptoms (i.e., optimising pain medication or blood pressure) were associated with being more active.

##### Physical environment of the hospital

The influence of the physical environment was mentioned many times, mostly by HCPs. Patient’ rooms were seen as inactivating as they offer insufficient space and lack suitable furniture, with a television set hanging right above the bed. HCPs suggested creating separate areas for sleeping and daytime activities, implementing measures to prevent spending too much time in bed, and providing every room with an exercise bike.

*H3015: ‘It’s very important to have a space where patients can eat or sit comfortably with their visitors. They should also be able to watch TV there, as currently patients have to lie in bed to watch TV.’*

Patients described the corridors as boring, cluttered, busy, and lacking places to sit or get coffee. Some described the need for a living room at the ward to socialise, eat, play games, or engage in craft work, while others did not perceive this need. Having an exercise room at the ward was also perceived as an enabler, although some participants did not know it was available. To make the environment of the ward and hospital more activating, patients and HCPs suggested creating more exercise facilities, places to sit, a coffee corner and possibilities to walk outside. They also described a need for organised activities such as craft work, musical and theatre performances. Moreover, as some patients were afraid of getting lost in the hospital, clearer signage, graphic symbols and walking routes were suggested to improve wayfinding.

##### Resources

A high workload and lack of time to promote PA were mentioned by many HCPs. This was often aggravated by a limited availability of staff, especially during weekends and evenings.

*H1006: ‘So in the end you don’t have enough staff to take everyone [patients] for a walk…’.*

On the other hand, having sufficient time to promote PA was described as an enabler. Furthermore, the availability of equipment (e.g., walking aids, wheelchairs, drain bag holders, lifting devices, portable oxygen tanks, anti-skid socks) and technology (e.g., virtual bike rides, gaming, virtual reality, interactive projections) were reported to be enablers.

#### 12. Social influence

Patients explained the importance of receiving encouragement and assistance from HCPs or visitors, because intrinsic motivation and other reasons to become active are often lacking during hospitalisation. Many described that they received less encouragement and assistance than they would have preferred.

*P0009: ‘Yes, you have to give people instructions. You don’t have to obey them, that’s up to you of course. But you need encouragement; subconsciously you know that it’s important, but you need to hear it from someone else and then it’s much more credible. Only then will it fully sink in.’*

On the other hand, patients and HCPs also described that HCPs sometimes provided more assistance than necessary. Additionally, HCPs reported that they should stimulate visitors more often to take patients for a walk, instead of sitting around.

#### 13. Emotion

No enablers emerged within this domain. Patients and HCPs reported that fear of falling or getting lost were barriers. They also described that patients may feel embarrassed to be seen walking around with a urinary catheter, drain or IV-pole.

*H3015: ‘Often patients are ashamed to walk with all these drains or a urinary catheter.’*

#### 14. Behavioural regulation

No barriers emerged within this domain. Patients and HCPs frequently mentioned the importance of providing information and education. Patients, visitors and HCPs need to be informed regarding the importance of staying active during hospitalisation, as well as regarding patients’ functional capabilities and the options available to stay active. Suggested modes of delivery were: face-to-face, brochures, posters, e-mail, TV and internet. Other strategies suggested to regulate behaviour were using mobility champions, using patient communication boards, providing regular training sessions, and regularly discussing functional capabilities within the team. HCPs also mentioned the importance of receiving practical skills training in safe patient handling and mobility. Furthermore, many HCPs discussed using technology (e.g., wearables, digital patient portals, tablets) as a strategy to promote PA behaviour. Physiotherapists highlighted the advantage of wearable activity monitors to assess patients’ PA levels and to set goals.

*H1014: ‘They always say: “Older adults, they can’t [deal with technology]”. I don’t know about that. I think that you should try it. I rather think that they don’t know what it’s like or how it works. I think they may surprise us.’*

## Discussion

To our knowledge, this is the first qualitative study to explore barriers and enablers to PA behaviour in older adults admitted to hospital with an acute medical illness, and to categorise them using the TDF. Our results offer an overview of barriers and enablers assigned to 11 of the 14 TDF domains, with the domain of *Environmental Context and Resources* being particularly elaborately described. Our findings highlight the complexity of influencing older adults’ PA behaviour during hospitalisation. Barriers and enablers were often reported as opposites, with the absence of a factor perceived as a barrier and its presence as an enabler. Although patients and HCPs reported many similar factors, HCPs reported a larger number of barriers and enablers as they perceive them from a broader perspective. While patients are hospitalised for a relatively short period and focus on their own illness, HCPs experience the hospital environment for a much longer period and provide care to many different patients. They take into account their patients’ as well as their own perspectives, hence perceiving more factors relating to care processes and organisational characteristics. The differences between patients’ and HCPs’ perspectives emphasise that both should be taken into account to gain a complete understanding of factors that influence older adults’ PA behaviour during hospitalisation. This setting-specific overview of barriers and enablers represents an initial step in developing, evaluating, and implementing theory-informed behaviour change interventions aimed at improving hospitalised older adults’ PA behaviour. It can assist clinicians and researchers in selecting modifiable factors that can be targeted in future interventions. Given that barriers and enablers may differ between settings and cultures, our overview can assist other settings in performing a setting-specific assessment to determine what needs to change to improve older adults’ PA behaviour in a different context. The current study yielded many barriers and enablers that will also be applicable to other contexts.

Within the domain of *Environmental Context and Resources*, our results confirm previous studies indicating that the hospital environment has an inactivating influence on patients [27, 49–51]. Care is organised around the hospital bed, with patients waiting for physician and nursing rounds, therapy services, visitors, and distribution of food or medication. Since patients are insufficiently aware of their daily schedule, they adopt a passive role and stay in their rooms, afraid to miss HCPs. Moreover, the physical environment and lack of available resources do not encourage patients to become active. Our study identified a number of factors within the domain of *Environmental Context and Resources* that were not identified in previous studies [17, 21–27], such as unnecessary waiting for HCPs (and ways to reduce this), the use of a bed or wheelchair to take patients to examinations, creating separate areas for sleeping and daytime activities, the availability of technology (e.g., interactive projections, virtual bike rides), attractive places (e.g., coffee corner, exercise room, garden) and activities (e.g., exhibitions, games, crafts, performances). To create a hospital culture aimed at improving older adults’ PA behaviour, our findings suggest that clinicians and researchers should consider reorganising care processes and organisational processes, restructuring the physical hospital environment and creating sufficient resources.

Our findings also suggest the importance of the domains of *Knowledge* and *Skills*. Awareness of the importance of PA, patients’ functional capabilities, and the available options to stay active are essential to encourage patients to become active during hospitalisation. Similarly, this awareness and having the skills to assist patients during PA are essential for HCPs, visitors and volunteers in order to encourage and assist patients. Doherty-King et al. supported this notion by describing how nurses considered their own abilities and experiences when deciding whether and how to mobilise patients [24]. Having knowledge and skills may positively influence other domains, such as *Intention* (e.g., motivation), *Beliefs about consequences* (e.g., believing that PA is necessary for recovery), *Social/Professional Role and Identity* (e.g., fewer patients adopting a ‘sick role’ and more HCPs perceiving responsibility to promote PA) and *Beliefs about capabilities* (e.g., nurses’ confidence about assisting patients). To improve knowledge and skills, many of the patients and HCPs would have liked to receive more information, education or practical skills training. Suggested strategies to accomplish this were using mobility champions, providing regular training sessions, using patient communication boards to visualize functional capabilities, and providing information face-to-face, via brochures or on TV. However, even when knowledge and skills are optimal, patients’ PA behaviour may still be impaired by other factors, such as pain or HCPs’ lack of time. Furthermore, many other modifiable factors were identified in the remaining domains, demonstrating the complexity of changing older adults’ PA behaviour during hospitalisation.

Given the large number of factors influencing the PA behaviour of hospitalised older adults, we recommend that clinicians and researchers develop, evaluate, and implement interventions targeted at multiple factors. Previous research suggests that such tailored multimodal interventions may be more effective than unimodal interventions [52]. In selecting which factors to target, clinicians and researchers should consider the impact and likelihood of changing patients’ PA behaviour and the ease of measurement when evaluating interventions. Selected factors can subsequently be linked to specific intervention functions and behaviour change techniques (i.e., active intervention components) [29, 32, 40, 53]. An available framework that may be useful to assist clinicians and researchers in selecting appropriate intervention functions is the Behaviour Change Wheel (BCW). The BCW offers a structured approach for the development of behaviour change interventions. It is a synthesis of 19 frameworks of behaviour change that is designed to link determinants of behaviour (using the TDF) to appropriate intervention functions and behaviour change techniques [32]. Additionally, the APEASE criteria (affordability, practicability, effectiveness and cost-effectiveness, acceptability, side-effects/safety, equity) could be useful in making strategic judgements in choosing the most appropriate intervention [32].

### Strengths and limitations

A strength of this study is the use of the TDF as a theoretical framework to categorise and understand barriers and enablers to PA behaviour in hospitalised older adults [29, 40]. Moreover, we have provided an in-depth description of factors that influence patients’ PA behaviour, by exploring barriers and enablers from a broad scope of perspectives of patients, nurses, physicians, and physiotherapists. For each patient, one of their HCPs was included as well, to explore different perspectives on the same situations. Lastly, almost all aspects of data collection and analysis were carried out by two researchers, with a third party available to solve disagreements.

We also acknowledge some limitations. While the TDF allows barriers and enablers to be explored from a broad perspective, it does not provide insight into the ways they interact with each other. Moreover, although an overview of theoretical definitions and component constructs was used to guide the coding process, it was difficult to differentiate between some TDF domains (e.g., *Knowledge* and *Beliefs about consequences*), as has also been reported by other studies [41, 54]. Furthermore, a limited number of demographic variables were collected for patients and HCPs. Providing additional patient characteristics (e.g., medication use or length of hospital stay) could have improved the understanding of patients’ PA behaviour. Moreover, information regarding HCPs’ own PA behaviour would have been valuable as this may have influenced HCPs’ thoughts and actions related to patients’ PA behaviour. Lastly, the majority of included HCPs were female with a relatively young median (IQR) age of 27 (24-33) years, and most physicians were resident physicians. Although this is thought to be an accurate representation of the population of HCPs in the Department of Internal Medicine, barriers and enablers may be perceived differently by younger and older, more experienced HCPs.

### Recommendations for future research

Further research is needed to develop and validate a TDF-based questionnaire that could facilitate a setting-specific assessment of barriers and enablers to PA behaviour in hospitalised older adults across all TDF domains. Moreover, few studies have investigated the efficacy or effectiveness of existing interventions aimed at improving the PA behaviour of older adults during hospitalisation [55–58]. Therefore, further studies are needed to develop, evaluate and implement theory-informed multimodal interventions that target setting-specific barriers and enablers to PA behaviour in hospitalised older adults.

## Conclusions

This study has yielded an overview of barriers and enablers to PA behaviour in hospitalised older adults admitted to a hospital with an acute medical illness, and has categorised them using the TDF. The large number of barriers and enablers identified highlights the complexity of influencing older adults’ PA behaviour during hospitalisation. We therefore recommend that clinicians and researchers develop interventions targeted at multiple factors. Our overview represents an initial step towards developing, evaluating, and implementing theory-informed behaviour change interventions to improve hospitalised older adults’ PA behaviour. It can assist clinicians and researchers in selecting modifiable factors that can be targeted in future interventions.

## Supplementary Information

**Additional file 1.** The consolidated criteria for reporting qualitative research (COREQ) checklist. A checklist of items that should be included in reports of qualitative research as recommended by the COREQ.

**Additional file 2.** Interview guide for patients. An interview guide with topics to discuss during semi-structured interviews with patients.

**Additional file 3.** Interview guide for healthcare professionals. An interview guide with topics to discuss during semi-structured interviews with healthcare professionals.

**Additional file 4.** The Theoretical Domains Framework (TDF) with definitions and component constructs. A list of fourteen TDF domains and their definitions and component constructs.

**Additional file 5.** Barriers and enablers to physical activity behaviour in older adults during hospital stay presented per TDF domain. A complete overview (coding tree) of the TDF coding of all barriers and enablers as reported by patients and healthcare professionals.

## Data Availability

The datasets used and/or analysed during the current study are available from the corresponding author on reasonable request.

## Abbreviations

PA: Physical activity
HCP: Healthcare professional
TDF: Theoretical Domains Framework
BCT: Behaviour change technique
MUMC+: Maastricht University Medical Centre
COREQ: Consolidated criteria for reporting Qualitative research
FAC: Functional ambulation categories
IQR: Interquartile range
BCW: Behaviour Change Wheel
APEASE: Affordability, practicability, effectiveness and cost-effectiveness, acceptability, side-effects/safety, equity
