# Supplementary material for: Barriers and enablers to physical activity behaviour in older adults during hospital stay: a qualitative study guided by the theoretical domains framework
Source: BMC Geriatr. 2022 Apr 10;22:314. doi: 10.1186/s12877-022-02887-x (PMC8994876; doi:10.1186/s12877-022-02887-x)
Supplement: Supplementary file 2 — Additional file 2. Interview guide for patients. An interview guide with topics to discuss during semi-structured interviews with patients. [file 12877_2022_2887_MOESM2_ESM.pdf]

Additional File 2 – Interview guide for patients

Introduction:

The purpose of this study is to explore barriers and enablers to physical activity during the hospital stay of older adults. Barriers are factors that reduce or negatively affect patients' engagement in physical activity, while enablers are factors that enhance or positively affect patients' engagement in physical activity. There are no right or wrong answers to these questions. Your opinion is important to this study, as it contributes to the improvement of physical activity levels of older adults during their hospital stay.

Topics:

- Could you describe your experience of being physically active during your hospital stay?
- Could you explain whether you have performed the same amount of physical activity during your hospital stay as you would have at home?
- How important is being physically active during your hospital stay to you?
- Which barriers to physical activity did you perceive during your hospital stay?
- Which enablers to physical activity did you perceive during your hospital stay?
- What should change according to you in order to increase patients' physical activity levels during their hospital stay?
- We have now elaborately discussed barriers and enablers to physical activity during your hospital stay. We have also discussed what should change in order to raise patients' physical activity levels. Out of all the possible improvements we have just discussed, please name three that would have the greatest impact in terms of raising patients' physical activity levels.
