# Supplementary material for: Barriers and enablers to physical activity behaviour in older adults during hospital stay: a qualitative study guided by the theoretical domains framework
Source: BMC Geriatr. 2022 Apr 10;22:314. doi: 10.1186/s12877-022-02887-x (PMC8994876; doi:10.1186/s12877-022-02887-x)
Supplement: Supplementary file 3 — Additional file 3. Interview guide for healthcare professionals. An interview guide with topics to discuss during semi-structured interviews with healthcare professionals. [file 12877_2022_2887_MOESM3_ESM.pdf]

Additional File 3 – Interview guide for healthcare professionals

Introduction:
The purpose of this study is to explore barriers and enablers to physical activity during the hospital stay of older adults. Barriers are factors that reduce or negatively affect patients' engagement in physical activity, while enablers are factors that enhance or positively affect patients' engagement in physical activity. We will also explore which factors have positively or negatively influenced your engagement in encouraging or assisting patients to be physically active. We will ask your opinion regarding the physical activity behaviour of [name of the patient], who is currently your patient. Furthermore, we will ask your opinion regarding the physical activity behaviour of the general population of patients aged 70 years or older who are admitted to the Department of Internal Medicine with an acute medical illness. There are no right or wrong answers to these questions. Your opinion is important to this study, as it contributes to the improvement of physical activity levels of older adults during their hospital stay.

Patient specific topics:
We will first discuss the hospital stay of [name of patient]. We have recently interviewed [name patient] and have explored his/her opinion.

- Which barriers to physical activity did [name of patient] perceive during his/her hospital stay?
- Which enablers to physical activity did [name of patient] perceive during his/her hospital stay?
- What should change according to you in order to enhance [name of patient]'s physical activity levels during his/her hospital stay?

General topics:
Now we will also discuss barriers and enablers to physical activity in the general population of patients aged 70 years or older admitted to the Department of Internal Medicine with an acute medical illness.

- Could you describe your experience of patients' physical activity behaviour during their hospital stay?
- How important is it that patients are physically active during their hospital stay?
- Which barriers to physical activity do patients perceive during their hospital stay?
- Which factors negatively influence your engagement in encouraging or assisting patients to be physically active?
- Which enablers to physical activity do patients perceive during their hospital stay?
- Which factors positively influence your engagement in encouraging or assisting patients to be physically active?
- What should change according to you in order to raise patients' physical activity levels during their hospital stay?
- We have now elaborately discussed barriers and enablers to physical activity during patients' hospital stay. We have also discussed what should change in order to raise patients' physical activity levels. Out of all the possible improvements we have just discussed, please name three that would have the greatest impact in terms of increasing patients' physical activity levels.
