# Supplementary material for: Barriers and enablers to physical activity behaviour in older adults during hospital stay: a qualitative study guided by the theoretical domains framework
Source: BMC Geriatr. 2022 Apr 10;22:314. doi: 10.1186/s12877-022-02887-x (PMC8994876; doi:10.1186/s12877-022-02887-x)
Supplement: Supplementary file 4 — Additional file 4. The Theoretical Domains Framework (TDF) with definitions and component constructs. A list of fourteen TDF domains and their definitions and component constructs. [file 12877_2022_2887_MOESM4_ESM.pdf]

Additional File 4. The Theoretical Domains Framework (TDF) with definitions and component constructs

TDF domain	TDF domain	Component constructs
**1. Knowledge**	An awareness of the existence of something	Knowledge (including knowledge of conditions/scientific rationale)<br>Procedural knowledge<br>Knowledge of task environment
**2. Skills**	An ability or proficiency acquired through practice	Skills<br>Skills development<br>Competence<br>Ability<br>Interpersonal skills<br>Practice<br>Skill assessment
**3. Social/Professional Role and Identity**	A coherent set of behaviors and displayed personal qualities of an individual in a social or work setting	Professional identity<br>Professional role<br>Social identity<br>Identity<br>Professional boundaries<br>Professional confidence<br>Group identity<br>Leadership<br>Organizational commitment

**4. Beliefs about Capabilities**	Acceptance of the truth, reality or validity about an ability, talent or facility that a person can put to constructive use	Self-confidence<br>Perceived competence<br>Self-efficacy<br>Perceived behavioral control<br>Beliefs<br>Self-esteem<br>Empowerment<br>Professional confidence
**5. Optimism**	The confidence that things will happen for the best or that desired goals will be attained	Optimism<br>Pessimism<br>Unrealistic optimism<br>Identity
**6. Beliefs about Consequences**	Acceptance of the truth, reality or validity about outcomes of a behavior in a given situation	Beliefs<br>Outcome expectancies<br>Characteristics of outcome expectancies<br>Anticipated regret<br>Consequents
**7. Reinforcement**	Increasing the probability of a response by arranging a dependent relationship, or contingency, between the response and a given stimulus	Rewards (proximal/distal, valued/not valued, probable/improbable)<br>Incentives<br>Punishment<br>Consequents<br>Reinforcement<br>Contingencies<br>Sanctions

**8. Intentions**	A conscious decision to perform a behavior or a resolve to act in a certain way	Stability of intentions<br>Stages of change model<br>Trans-theoretical model and stages of change
**9. Goals**	Mental representations of outcomes or end states that an individual wants to achieve	Goals (distal/proximal)<br>Goal priority<br>Goal/target setting<br>Goals (autonomous/controlled)<br>Action planning<br>Implementation intention
**10. Memory, Attention and Decision Process**	The ability to retain information, focus selectively on aspects of the environment and choose between two or more alternatives	Memory<br>Attention<br>Attention control<br>Decision making<br>Cognitive overload/tiredness
**11. Environmental Context & Resources**	Any circumstance of a person's situation or environment that discourages or encourages the development of skills and abilities, independence, social competence and adaptive behavior	Environmental stressors<br>Resources/material resources<br>Organizational culture/climate<br>Salient events/critical incidents<br>Person × environment interaction<br>Barriers and facilitators
**12. Social Influences**	Those interpersonal processes that can cause individuals to change their thoughts, feelings or behaviors	Social pressure<br>Social norms<br>Group conformity<br>Social comparisons<br>Group norms<br>Social support

		Power<br>Intergroup conflict<br>Alienation<br>Group identity<br>Modelling
**13. Emotion**	A complex reaction pattern, involving experiential, behavioral, and physiological elements, by which the individual attempts to deal with a personally significant matter or event	Fear<br>Anxiety<br>Affect<br>Stress<br>Depression<br>Positive/negative affect<br>Burn-out
**14. Behavioral Regulation**	Anything aimed at managing or changing objectively observed or measured actions.	Self-monitoring<br>Breaking habit<br>Action planning

TDF = Theoretical Domains Framework

Reference:

Atkins L, Francis J, Islam R, O'Connor D, Patey A, Ivers N, et al. A guide to using the Theoretical Domains Framework of behaviour change to investigate implementation problems. Implement Sci. 2017;12(1):77
