# Supplementary material for: Barriers and enablers to physical activity behaviour in older adults during hospital stay: a qualitative study guided by the theoretical domains framework
Source: BMC Geriatr. 2022 Apr 10;22:314. doi: 10.1186/s12877-022-02887-x (PMC8994876; doi:10.1186/s12877-022-02887-x)
Supplement: Supplementary file 5 — Additional file 5. Barriers and enablers to physical activity behaviour in older adults during hospital stay presented per TDF domain. A complete overview (coding tree) of the TDF coding of all barriers and enablers as reported by patients and healthcare professionals. [file 12877_2022_2887_MOESM5_ESM.docx]

**Additional File 5.** Barriers and enablers to physical activity behaviour in older adults during hospital stay presented per TDF domain

|  | **Barriers** |  | **HCP** | | | **Enablers** |  | **HCP** | | |
| --- | --- | --- | --- | --- | --- | --- | --- | --- | --- | --- |
|  |  | **P** | **N** | **PH** | **PT** |  | **P** | **N** | **PH** | **PT** |
| **1. Knowledge** | | | | | | | | | | |
|  | **HCPs' knowledge** |  |  |  |  | **HCPs' knowledge** |  |  |  |  |
|  | Lack of insight into the amount of PA performed by patients |  |  | x | x | Knowledge about patients' functional capabilities | x | x | x |  |
|  | Lack of knowledge about patients' functional capabilities  during hospitalisation |  | x | x | x | **Patients' knowledge** |  |  |  |  |
|  | Lack of knowledge about patients' functional capabilities prior to hospital admission | x |  | x |  | Knowledge about their own functional capabilities |  |  |  | x |
|  | Lack of knowledge about the importance of PA |  |  |  | x | Expectations regarding PA during hospitalisation are clear prior to admission |  |  |  | x |
|  | Lack of knowledge about the PA options |  |  | x |  | **Visitors' knowledge** |  |  |  |  |
|  | Lack of knowledge about walking aids and medical devices |  |  | x | x | Knowledge about patients’ functional capabilities |  | x |  | x |
|  | **Patients' knowledge** |  |  |  |  |  |  |  |  |  |
|  | Lack of knowledge about their own functional capabilities | x | x | x | x |  |  |  |  |  |
|  | Lack of knowledge about opportunities for being physically active despite restrictive medical devices (intravenous pole, urinary catheter, oxygen) | x | x | x |  |  |  |  |  |  |
|  | Lack of knowledge about the expectations regarding PA during hospitalisation |  |  |  | x |  |  |  |  |  |
|  | Lack of knowledge about the importance of PA | x | x | x | x |  |  |  |  |  |
|  | Lack of knowledge about the PA options | x | x | x | x |  |  |  |  |  |
|  | Lack of knowledge about the possibility to eat in a communal dining room | x |  |  |  |  |  |  |  |  |
|  | **Visitors' knowledge** |  |  |  |  |  |  |  |  |  |
|  | Lack of knowledge about patients' functional capabilities |  | x |  | x |  |  |  |  |  |
|  | Lack of knowledge about the expectations regarding PA during hospitalisation |  | x |  | x |  |  |  |  |  |
|  | Lack of knowledge about the importance of PA |  | x |  |  |  |  |  |  |  |
|  | Lack of knowledge about the PA options | x | x |  |  |  |  |  |  |  |
| **2. Skills** | | | | | | | | | | |
|  | **HCPs' skills** |  |  |  |  | **HCPs' skills** |  |  |  |  |
|  | Lack of skills to assist patients with PA | x | x |  | x | Nurses having skills to assist patients with PA |  | x |  |  |
|  | Communicative approach | x |  |  |  | **Patients' skills** |  |  |  |  |
|  | **Patients' skills** |  |  |  |  | Being independent for PA |  |  |  | x |
|  | Being dependent on others for PA | x | x | x | x |  |  |  |  |  |
|  | Fall risk | x | x | x | x |  |  |  |  |  |
|  | **Visitors' skills** |  |  |  |  |  |  |  |  |  |
|  | Lack of skills to assist patients with PA |  |  | x | x |  |  |  |  |  |
|  | **Volunteers' skills** |  |  |  |  |  |  |  |  |  |
|  | Lack of skills to assist patients with PA |  | x |  |  |  |  |  |  |  |
| **3. Social / Professional Role & Identity** | | | | | | | | | | |
|  | **HCP's role** |  |  |  |  | **HCPs role** |  |  |  |  |
|  | Lack of openness to change |  | x |  |  | Nurses' role |  |  |  |  |
|  | Lack of perceived responsibility to encourage and assist patients with PA |  | x | x | x | Assessing functional capabilities |  | x | x | x |
|  | Nurses' lack of authority |  | x |  |  | Providing encouragement and assistance |  | x |  | x |
|  | **Patients' role** |  |  |  |  | Providing patients with information on the importance of PA during hospitalisation |  | x | x |  |
|  | Sick role |  |  |  |  | Providing walking aids |  | x | x |  |
|  | Patients adopting a passive and dependent attitude |  | x |  | x | Training other nurses in safe patient handling and mobility |  | x |  |  |
|  | The idea that older adults don't need to be active | x | x | x | x | Physicians' role |  |  |  |  |
|  | The idea that PA does not need to be resumed until patients have returned home | x | x |  |  | Providing patients with information on the importance of PA during hospitalisation |  | x | x |  |
|  | The idea that patients should remain in bed | x | x | x | x | Referring patients to therapy services |  | x | x | x |
|  |  |  |  |  |  | Status of a physician |  | x | x | x |
|  |  |  |  |  |  | Physiotherapists' role |  |  |  |  |
|  |  |  |  |  |  | Assessing fall risk and fear of falling |  |  | x | x |
|  |  |  |  |  |  | Assessing functional capabilities and risk of low PA levels |  | x | x | x |
|  |  |  |  |  |  | Coaching patients, visitors and HCPs | x | x | x | x |
|  |  |  |  |  |  | Educating other HCPs on the importance of PA during hospitalisation |  |  |  | x |
|  |  |  |  |  |  | Instructing group exercise classes |  | x |  | x |
|  |  |  |  |  |  | Providing encouragement and assistance |  | x |  | x |
|  |  |  |  |  |  | Providing patients with information on the importance of PA during hospitalisation | x | x | x | x |
|  |  |  |  |  |  | Providing supervision in exercise room | x |  |  | x |
|  |  |  |  |  |  | Providing walking aids |  |  | x | x |
|  |  |  |  |  |  | Status of a physiotherapist |  | x |  |  |
|  |  |  |  |  |  | Encouraging prevention |  |  |  | x |
|  |  |  |  |  |  | Training HCPs in safe patient handling and mobility |  | x |  | x |
|  |  |  |  |  |  | Promoting PA behaviour is perceived as a multidisciplinary team effort |  |  | x | x |
|  |  |  |  |  |  | The multidisciplinary team is dedicated to promoting PA behaviour |  | x |  | x |
| **4. Beliefs about Capabilities** | | | | | | | | | | |
|  | **Patients' capabilities** |  |  |  |  | **Patients’ capabilities** |  |  |  |  |
|  | Lack of confidence in one’s own capabilities | x |  | x | x | Having confidence in one’s own capabilities | x |  |  | x |
| **5. Optimism** | | | | | | | | | | |
|  | - |  |  |  |  | Patients having a positive attitude | x |  | x | x |
| **6. Beliefs about Consequences** | | | | | | | | | | |
|  | Believing that PA results in negative consequences |  |  |  |  | Believing that PA results in positive outcomes |  |  |  |  |
|  | Patients cannot be reached when they are off the ward |  | x |  |  | Believing that PA is needed for recovery | x |  | x |  |
|  |  |  |  |  |  | Believing that PA will improve physical fitness | x |  |  |  |
| **7. Reinforcement** | | | | | | | | | | |
|  | - |  |  |  |  | - |  |  |  |  |
| **8. Intentions** | | | | | | | | | | |
|  | **HCPs' intention** |  |  |  |  | **HCPs' intention** |  |  |  |  |
|  | Lack of motivation to encourage or assist patients to be active |  | x | x | x | Motivation to encourage and assist patients to be active |  | x |  |  |
|  | **Patients' intention** |  |  |  |  | Having a dedicated team |  | x |  | x |
|  | Lack of intention to leave the room or ward | x |  |  | x | **Patients' intention** |  |  |  |  |
|  | Lack of motivation to be active | x | x | x | x | Motivation to be active | x | x | x | x |
|  | Being lazy | x | x | x |  |  |  |  |  |  |
| **9. Goals** | | | | | | | | | | |
|  | Not having a reason to be active | x | x | x | x | Goal-setting | x | x | x | x |
|  |  |  |  |  |  | Wanting to experience the positive effects of PA | x |  |  |  |
|  |  |  |  |  |  | Wanting to recover | x |  | x |  |
|  |  |  |  |  |  | Wanting to go home | x | x | x | x |
|  |  |  |  |  |  | Wanting to improve physical fitness | x |  |  |  |
|  |  |  |  |  |  | Wanting to regain patients’ self-confidence | x |  |  |  |
|  |  |  |  |  |  | Wanting to prevent the negative effects of inactivity |  |  |  |  |
|  |  |  |  |  |  | Wanting to prevent being dependent on others | x | x |  |  |
|  |  |  |  |  |  | Wanting to prevent complications |  |  |  |  |
|  |  |  |  |  |  | Pain and stiffness | x |  |  |  |
|  |  |  |  |  |  | Functional decline | x | x | x | x |
|  |  |  |  |  |  | Pressure ulcers | x | x | x |  |
|  |  |  |  |  |  | Pulmonary complications |  | x |  |  |
|  |  |  |  |  |  | Deep vein thrombosis | x |  | x |  |
|  |  |  |  |  |  | Mortality | x |  |  |  |
|  |  |  |  |  |  | Wanting to feel satisfied | x |  |  |  |
|  |  |  |  |  |  | Wanting to prevent boredom | x | x | x | x |
|  |  |  |  |  |  | Wanting some distraction | x |  | x |  |
|  |  |  |  |  |  | Wanting privacy (by leaving the room) |  |  | x | x |
|  |  |  |  |  |  | Wanting to smoke |  | x | x |  |
| **10. Memory, Attention and Decision Process** | | | | | | | | | | |
|  | **HCPs’ MADP** |  |  |  |  | - |  |  |  |  |
|  | Discrepant views about the priority of promoting PA within a team |  | x | x | x |  |  |  |  |  |
|  | Prioritisation forced by high patient acuity |  | x |  |  |  |  |  |  |  |
|  | **Patients’ MADP** |  |  |  |  |  |  |  |  |  |
|  | Not wanting to bother HCPs |  |  |  | x |  |  |  |  |  |
|  | PA does not have priority during hospitalisation | x | x | x | x |  |  |  |  |  |
| **11. Environmental Context & Resources** | | | | | | | | | | |
|  | **Care processes and organisational characteristics** |  |  |  |  | **Care processes and organisational characteristics** |  |  |  |  |
|  | Taking patients to examinations in a bed or wheelchair |  | x | x | x | Assessment of patients’ functional capabilities |  | x | x | x |
|  | Daily schedule |  |  |  |  | Avoiding the use of a bed or wheelchair to take patients to examinations |  | x | x |  |
|  | Busy daily schedule limits time for PA | x | x | x | x | Daily schedule |  |  |  |  |
|  | Lacking structure and insight into one’s own daily schedule | x |  |  | x | Care processes are scheduled at predetermined times, creating time for rest and PA | x |  |  | x |
|  | Limited possibilities for PA in evenings and weekends | x | x | x | x | Daily schedule is structured and enables PA |  | x |  |  |
|  | Most PA is performed in the morning |  |  |  | x | Organised activities are provided throughout the whole day | x | x | x | x |
|  | Waiting | x |  |  | x | PA behaviour is promoted during evenings and weekends |  | x |  | x |
|  | For care or HCPs | x |  | x | x | Waiting |  |  |  |  |
|  | For nursing assistance | x | x | x | x | Reducing unnecessary waiting for care or HCPs | x |  | x | x |
|  | For physician rounds | x | x | x | x | Waiting too long for nurses' assistance makes patients walk to the bathroom alone | x |  |  |  |
|  | For visitors | x |  |  |  | Eating and drinking |  |  |  |  |
|  | Eating and drinking |  |  |  |  | Eating in a communal dining room | x | x | x | x |
|  | Food and drinks are always brought to the patients' room | x | x | x | x | Eating out of bed | x | x |  |  |
|  | Lack of opportunities to eat together in a communal dining room | x | x | x |  | Food and drinks are only brought to the patients’ room for patients that are not capable of eating in a communal dining room | x | x |  | x |
|  | Patients eating in bed |  | x |  | x | Having patients assist in setting the table | x |  |  | x |
|  | Lack of well-established referral pathways to therapy services |  | x | x | x | Availability of facilities where patients can get their own drinks | x | x | x | x |
|  | Load - endurance (taking patients' physical capabilities insufficiently into account / leaving patients out of bed for too long) | x | x | x | x | Multidisciplinary collaboration and communication |  | x |  | x |
|  | Multidisciplinary collaboration and communication |  |  | x | x | Interdisciplinary communication |  | x | x | x |
|  | Interdisciplinary communication |  | x | x | x | Physician communicates restrictions  regarding PA to the multidisciplinary team |  | x |  |  |
|  | Communication between nurses |  | x |  | x | Communication between nurses |  | x | x |  |
|  | High administrative burden |  | x | x |  | PA behaviour is promoted by the whole multidisciplinary team |  |  | x | x |
|  | Difficult retrieval of information from electronic medical records |  | x |  |  | Documentation about PA and functional mobility in electronic medical record |  | x | x | x |
|  | Multidisciplinary consultation meetings |  | x |  | x | Multidisciplinary consultation meetings |  | x | x | x |
|  | Organisational characteristics |  |  |  |  | Organisational characteristics |  |  |  |  |
|  | Hospital culture facilitates sedentary behaviour |  |  | x | x | Evaluating and optimising care processes |  |  |  | x |
|  | Lack of hospital policy regarding promotion of PA behaviour |  | x |  | x | By involving consultants |  |  |  | x |
|  | Lack of enforcement of hospital policy regarding promotion of PA behaviour |  | x |  | x | Through multidisciplinary quality improvement projects |  |  |  | x |
|  | Lack of openness to change within hospital culture |  | x |  |  | By optimising HCPs’ roles within multidisciplinary teams |  |  |  | x |
|  | Restrictions imposed on volunteers regarding assisting patients with PA |  | x |  |  | Policy regarding PA promotion |  | x | x | x |
|  | Physician rounds - Not discussing PA behaviour during physician rounds | x |  | x | x | Management supports hospital policy regarding promotion of PA behaviour |  | x |  | x |
|  | Physiotherapy |  |  |  |  | Mission statement of the organisation includes the importance of PA |  |  |  | x |
|  | Not providing tailored, individualised care |  |  |  | x | Policy aimed at minimising physical restraint and freedom restricting measures |  | x | x | x |
|  | Patients being treated by too many different physiotherapists during their hospital stay |  |  |  | x | Policy allowing portable oxygen tanks in the patients' room |  |  |  | x |
|  | Providing bed-centred care |  |  |  | x | Policy to encourage visitors not to sit around the bed |  | x | x | x |
|  | Using medical devices that hinder PA (drains, urinary catheters, wheelchair, IV-poles, oxygen) | x | x | x | x | Physical restraint and freedom restricting measures |  |  |  |  |
|  | Using physical restraint and freedom restricting measures | x | x | x | x | Facilitating PA despite using physical restraints and freedom restricting measures |  | x |  | x |
|  | **Patient-related factors** |  |  |  |  | Minimising the use of physical restraint and freedom restricting measures |  | x | x |  |
|  | Older adults (higher age) | x | x | x | x | Physician rounds |  |  |  |  |
|  | Medical factors |  |  |  |  | Assessment of functional capabilities during physician rounds |  |  |  | x |
|  | Comorbidities | x | x | x |  | Discussing PA behaviour during physician rounds |  | x | x | x |
|  | Cognitive problems |  | x | x | x | Patients going to physicians instead of the other way around |  |  |  | x |
|  | Delirium | x | x | x | x | Physician rounds at set times | x |  |  | x |
|  | Dementia |  | x |  |  | Physiotherapist joining physician rounds |  | x | x |  |
|  | Obesity |  | x |  | x | Physiotherapy |  |  |  |  |
|  | Visual and hearing impairments | x |  |  | x | Assessing functional capabilities and risk of low PA levels |  | x | x | x |
|  | Deterioration of physical functioning prior to hospital admission | x |  | x |  | Being referred to physiotherapy | x | x |  |  |
|  | High level of complexity of medical care |  | x |  | x | Coaching patients, visitors, and HCPs | x | x | x | x |
|  | Illness and symptoms | x | x | x | x | Improving patients' functional capabilities and PA levels | x | x |  | x |
|  | Diarrhoea | x |  |  |  | Physiotherapist is present at the ward at predetermined times |  | x |  | x |
|  | Dizziness | x | x | x |  | Providing group exercise classes |  | x |  | x |
|  | Dyspnoea |  | x | x | x | Supervising exercise hours | x |  |  | x |
|  | Fatigue | x | x | x |  | Providing Function-focused care | x | x | x | x |
|  | Hypotension | x |  | x |  | Providing patients with suitable walking aids |  | x | x | x |
|  | Nausea |  | x |  |  | Providing tailored, individualised care |  |  | x | x |
|  | Oedema |  | x |  |  | Assessing patients’ barriers to PA behaviour |  |  | x |  |
|  | Pain | x | x | x |  | Load - endurance (taking patients' physical capabilities into account) |  | x | x | x |
|  | Poor balance | x | x | x |  | Using well-established referral pathways to therapy  services |  | x | x | x |
|  | Reduced exercise capacity | x | x |  |  |  |  |  |  |  |
|  | Weakness | x | x | x |  | **Patient-related factors** |  |  |  |  |
|  | Medical factors necessitating bedrest |  | x | x |  | Medical factors |  |  |  |  |
|  | Medication | x |  | x |  | Having to go to the bathroom | x |  |  |  |
|  | **Physical environment of the hospital** |  |  |  |  | Recovery | x | x |  |  |
|  | Patient room | x |  | x | x | Symptom management |  |  |  |  |
|  | Lack of suitable furniture |  | x | x | x | Optimising pain medication | x | x | x | x |
|  | Lack of space | x | x | x | x | Regular blood pressure checks |  | x |  |  |
|  | Multi-person room does not facilitate rest | x |  |  |  | **Physical environment of the hospital** |  |  |  |  |
|  | Television set hanging right above the bed | x | x | x | x | Patient rooms |  |  |  |  |
|  | Ward environment |  | x | x | x | Availability of suitable furniture | x | x |  | x |
|  | Uninteresting corridors | x | x | x | x | Availability of an en-suite bathroom | x | x |  | x |
|  | Cluttered, busy corridors | x |  |  | x | Availability of an exercise bike | x |  |  | x |
|  | Communal ‘living room’ (with or without organised activities) | x | x | x | x | Availability of separate areas for sleeping and daytime activities | x | x | x | x |
|  | Lack of exercise rooms or exercise facilities | x | x | x | x | Availability of sufficient space |  | x |  | x |
|  | Lack of facilities to which patients can walk to get a drink | x | x | x | x | Measures discouraging lying in bed  (except when sleeping or resting) |  | x | x | x |
|  | Lack of places to rest |  | x |  | x | Multi-person room | x |  |  | x |
|  | Poor wayfinding | x | x | x | x | Single room | x |  |  | x |
|  | **Resources** |  |  |  |  | Television set not hanging right above the bed |  | x | x | x |
|  | High workload for nurses (lack of time) | x | x | x | x | Ward environment |  | x | x | x |
|  | Limited availability of walking aids |  | x |  | x | Availability of a clutter-free corridor | x |  |  | x |
|  | Limited staffing | x | x |  | x | Availability of a coffee corner | x |  |  |  |
|  | During evenings and weekends | x | x | x | x | Availability of a communal ‘living room’ | x | x | x | x |
|  | Patients’ personal belongings (clothing, shoes, walking aid) not present at the hospital | x | x | x | x | To be used for organised activities | x | x | x | x |
|  |  |  |  |  |  | To be used as a dining room |  | x | x |  |
|  |  |  |  |  |  | Availability of an exercise room or exercise facilities | x | x | x | x |
|  |  |  |  |  |  | Availability of places to sit in the corridor | x | x | x | x |
|  |  |  |  |  |  | Availability of walking routes |  |  |  | x |
|  |  |  |  |  |  | Cosy atmosphere | x | x | x | x |
|  |  |  |  |  |  | Hospital | x | x | x | x |
|  |  |  |  |  |  | Cosy atmosphere |  |  |  | x |
|  |  |  |  |  |  | Availability of a coffee corner |  | x |  | x |
|  |  |  |  |  |  | Availability of attractive places | x | x | x | x |
|  |  |  |  |  |  | Availability of walking routes | x | x |  | x |
|  |  |  |  |  |  | Outside | x |  |  |  |
|  |  |  |  |  |  | Availability of a garden |  |  |  | x |
|  |  |  |  |  |  | Wayfinding |  |  |  |  |
|  |  |  |  |  |  | Availability of clear signage | x |  |  | x |
|  |  |  |  |  |  | Availability of graphic symbols outside patient rooms to improve recognition |  | x |  | x |
|  |  |  |  |  |  | Availability of walking routes | x | x |  | x |
|  |  |  |  |  |  | **Resources** |  |  |  |  |
|  |  |  |  |  |  | Availability of equipment |  |  |  |  |
|  |  |  |  |  |  | Anti-skid socks |  |  |  | x |
|  |  |  |  |  |  | Drain bag holders |  |  |  | x |
|  |  |  |  |  |  | Exercise and fitness equipment |  |  |  | x |
|  |  |  |  |  |  | Lifting devices |  | x |  |  |
|  |  |  |  |  |  | Portable oxygen tanks |  |  |  | x |
|  |  |  |  |  |  | Walking aids | x | x | x | x |
|  |  |  |  |  |  | Wheelchairs |  | x | x |  |
|  |  |  |  |  |  | Availability of meaningful activities |  |  |  |  |
|  |  |  |  |  |  | Bingo |  |  |  | x |
|  |  |  |  |  |  | Exhibitions | x | x | x |  |
|  |  |  |  |  |  | Games | x | x |  | x |
|  |  |  |  |  |  | Lectures | x |  |  |  |
|  |  |  |  |  |  | Craft work | x | x |  | x |
|  |  |  |  |  |  | Movies |  |  |  | x |
|  |  |  |  |  |  | Organised exercise classes | x | x | x | x |
|  |  |  |  |  |  | Organised group activities or recreational therapy | x | x | x | x |
|  |  |  |  |  |  | Performances (musical, theatre) | x | x | x | x |
|  |  |  |  |  |  | Reading (availability of newspapers, books) | x | x | x | x |
|  |  |  |  |  |  | Staff availability |  | x |  | x |
|  |  |  |  |  |  | Availability of an activity counsellor in the communal room |  |  | x |  |
|  |  |  |  |  |  | Availability of volunteers | x | x | x | x |
|  |  |  |  |  |  | Availability of sufficient time – workload is not too high |  | x | x | x |
|  |  |  |  |  |  | Patients’ personal belongings (clothing, shoes, walking aid) are present at the hospital |  | x |  | x |
|  |  |  |  |  |  | Technology |  |  |  |  |
|  |  |  |  |  |  | Gaming |  |  |  | x |
|  |  |  |  |  |  | Virtual bike rides |  | x |  | x |
|  |  |  |  |  |  | Interactive projections |  | x |  | x |
|  |  |  |  |  |  | Virtual Reality |  | x |  |  |
| **12. Social influences** | | | | | | | | | | |
|  | **HCPs’ influence** |  |  |  |  | **HCPs’ influence** |  |  |  |  |
|  | Lack of encouragement and assistance | x |  | x | x | Involving visitors in promoting PA behaviour |  | x | x | x |
|  | Providing more care than necessary |  |  | x | x | Patient - HCP relationship based on trust | x | x | x |  |
|  | **Patients’ influence** |  |  |  |  | Positive patient - HCP interaction | x | x | x | x |
|  | No need for social interaction | x |  |  |  | Presence of a physician on the ward |  |  | x | x |
|  | Not following HCPs’ advice |  | x | x | x | Providing encouragement and assistance | x | x | x | x |
|  | **Visitors’ influence** |  |  |  |  | To go outside | x | x |  |  |
|  | Lack of encouragement and assistance | x | x | x | x | To go to examinations, treatment or activities elsewhere in the hospital | x | x | x |  |
|  | Discrepancy between patients' and HCPs' expectations regarding the amount of care | x |  |  | x | To improve wayfinding | x |  |  | x |
|  |  |  |  |  |  | To patients lacking motivation to be active | x | x |  |  |
|  |  |  |  |  |  | To patients who are dependent on others during PA | x | x | x | x |
|  |  |  |  |  |  | To patients with cognitive impairments | x |  |  | x |
|  |  |  |  |  |  | To patients with fear of walking |  | x |  |  |
|  |  |  |  |  |  | **Other patients’ influence** |  |  |  |  |
|  |  |  |  |  |  | Providing encouragement and assistance | x |  |  | x |
|  |  |  |  |  |  | **Patients’ influence** |  |  |  |  |
|  |  |  |  |  |  | Following HCPs’ advice |  |  | x | x |
|  |  |  |  |  |  | Wanting social interaction | x | x | x | x |
|  |  |  |  |  |  | **Visitors’ influence** |  |  |  |  |
|  |  |  |  |  |  | Providing encouragement and assistance | x | x | x | x |
| **13. Emotion** | | | | | | | | | | |
|  | Embarrassment | x | x |  | x | - |  |  |  |  |
|  | Fear (of falling or getting lost) | x | x | x | x |  |  |  |  |  |
|  | Self-pity | x |  |  |  |  |  |  |  |  |
| **14. Behavioural Regulation** | | | | | | | | | | |
|  | - |  |  |  |  | Providing information |  |  |  |  |
|  |  |  |  |  |  | Providing information on patients' functional capabilities |  |  |  |  |
|  |  |  |  |  |  | Providing HCPs with information on patients' functional capabilities |  |  |  | x |
|  |  |  |  |  |  | By using patient communication boards |  | x | x | x |
|  |  |  |  |  |  | By discussing case studies within the team |  |  |  | x |
|  |  |  |  |  |  | Using mobility champions |  | x |  |  |
|  |  |  |  |  |  | Providing patients and visitors with information on patients’ functional capabilities | x | x | x | x |
|  |  |  |  |  |  | Delivered face-to-face | x | x | x | x |
|  |  |  |  |  |  | Delivered via brochures |  | x |  | x |
|  |  |  |  |  |  | Delivered via short videos (television - internet) |  |  |  | x |
|  |  |  |  |  |  | By using patient communication boards | x | x | x | x |
|  |  |  |  |  |  | Providing patients with information on their  functional capabilities despite being attached  to restrictive medical devices |  | x | x | x |
|  |  |  |  |  |  | Providing information on the importance of PA |  |  |  |  |
|  |  |  |  |  |  | Providing information to HCPs |  |  | x |  |
|  |  |  |  |  |  | Providing regular training sessions |  | x | x | x |
|  |  |  |  |  |  | Providing information to patients and visitors | x | x | x | x |
|  |  |  |  |  |  | Delivered face-to-face | x | x | x | x |
|  |  |  |  |  |  | Delivered via brochures |  | x | x | x |
|  |  |  |  |  |  | Delivered via posters | x | x | x | x |
|  |  |  |  |  |  | Delivered via short videos (television - internet) |  | x |  | x |
|  |  |  |  |  |  | Providing patients and visitors with information (during or prior to admission) to manage expectations of PA during hospitalisation | x | x | x | x |
|  |  |  |  |  |  | Delivered face-to-face | x | x | x | x |
|  |  |  |  |  |  | Delivered via email |  | x |  |  |
|  |  |  |  |  |  | Delivered via general practitioner |  |  |  | x |
|  |  |  |  |  |  | Delivered via media |  |  |  | x |
|  |  |  |  |  |  | Delivered via short videos (television - internet) |  |  |  | x |
|  |  |  |  |  |  | Instructing patients to bring their own walking aid |  |  | x | x |
|  |  |  |  |  |  | Providing patients with information on the available PA options | x | x | x |  |
|  |  |  |  |  |  | Delivered via calendar showing weekly activities |  |  |  | x |
|  |  |  |  |  |  | Providing HCPs with a ‘safe patient handling and mobility’ skills training |  | x |  | x |
|  |  |  |  |  |  | Using maps to improve patients' wayfinding |  |  |  | x |
|  |  |  |  |  |  | Using a screening tool to identify patients at risk for  low PA levels or functional decline |  |  |  | x |
|  |  |  |  |  |  | Using goal-setting | x | x |  |  |
|  |  |  |  |  |  | Using strategies to reduce unnecessary waiting |  |  |  |  |
|  |  |  |  |  |  | Using a buzzer |  |  |  | x |
|  |  |  |  |  |  | Using a daily schedule | x |  | x | x |
|  |  |  |  |  |  | Using an 'I am available’-door-hanger |  |  |  | x |
|  |  |  |  |  |  | Using technology | x | x | x | x |
|  |  |  |  |  |  | Using a digital patient portal |  |  |  | x |
|  |  |  |  |  |  | Using a tablet / laptop / computer | x | x |  | x |
|  |  |  |  |  |  | Monitoring physical activity (wearables) | x |  | x |  |

TDF = Theoretical Domains Framework; PA = physical activity; HCP = healthcare professional; P = Patient; N = nurse; PH = physician; PT = physiotherapist; MADP = Memory, Attention and Decision Process.
